# Black patients with multiple myeloma have better survival than white patients when treated equally: a matched cohort study

**DOI:** 10.1038/s41408-022-00633-5

**Published:** 2022-02-24

**Authors:** Jing Dong, Zhuping Garacci, Christopher Staffi Buradagunta, Anita D’Souza, Meera Mohan, Ashley Cunningham, Siegfried Janz, Binod Dhakal, Aaron P. Thrift, Parameswaran Hari

**Affiliations:** 1 Division of Hematology Oncology, Department of Medicine, Medical College of Wisconsin, Milwaukee, WI USA; 2 Medical College of Wisconsin Cancer Center, Milwaukee, WI USA; 3 Center for Advancing Population Science, Medical College of Wisconsin, Milwaukee, WI USA; 4 Center for International Blood and Marrow Transplant Research, Medical College of Wisconsin, Milwaukee, WI USA; 5 Department of Pathology, Medical College of Wisconsin, Milwaukee, WI USA; 6 Section of Epidemiology and Population Sciences, Department of Medicine, and Dan L Duncan Comprehensive Cancer Center, Baylor College of Medicine, Houston, TX USA

**Keywords:** Risk factors, Epidemiology

## Abstract

We assessed differences in survival between non-Hispanic black (NHB) and non-Hispanic white (NHW) patients with multiple myeloma (MM), and the sequential effects of patient characteristics, and diagnosis and treatment-related factors on the survival disparity using data from 3319 NHB and 20,831 NHW MM patients in the SEER-Medicare (1999–2017) database. Four sets of 3319 NHWs were matched sequentially to the same set of 3319 NHBs, based on demographics (age, sex, year of diagnosis, marital status, and SEER site), socioeconomic status (SES, demographics plus SES), presentation factors (SES variables plus comorbidity), and treatment factors (presentation variables plus antimyeloma therapies). We found NHBs were less likely to receive treatment than NHWs even among patients matched for demographics, SES, and comorbidities. The absolute difference in 5-year survival between NHBs and NHWs was not significant in the demographics match (0.6%; *P* = 0.30) and remained non-significant after matching for SES (1.4%, *P* = 0.17). When matching for presentation, NHBs had significantly longer 5-year survival than NHWs (absolute difference = 3.8%, *P* = 0.003). Additional matching on treatment-related factors further enlarged the racial difference in 5-year survival to 4.6% (*P* < 0.001). Our findings reinforce the importance of equitable access to effective treatment modalities to further improve the survival of NHB patients with MM.

## Introduction

Multiple myeloma (MM), characterized by the proliferation of clonal plasma cells in the bone marrow, is the second most common hematologic malignancy in the US [1, 2]. Non-Hispanic blacks (NHBs) are disproportionately affected by MM, with 2- to 3-fold higher incidence, younger age of onset and more than double the mortality compared to non-Hispanic whites (NHWs) [2–6]. In addition, NHBs are reported to have a lower utilization rate of novel therapeutic agents (e.g., proteasome inhibitors (PIs) and immunomodulatory drugs (IMiDs)) and autologous stem cell transplantation (ASCT) than NHWs. However, despite the lower treatment rates in NHBs, whether this translates into poorer outcomes remains unclear [7–12]. Most Surveillance, Epidemiology and End Results (SEER)-based analyses reported NHBs had either similar or better overall survival (OS), and better myeloma-specific survival than NHWs after adjusting for demographic factors, comorbidities and/or treatment [7–9]; while data from the Multiple Myeloma Research Foundation CoMMpass study showed a persistent inferior OS in NHBs [10]. These studies applied model-based methods which, when fitted to the entire population, give disproportionate weighting to the larger NHW population [13]. In addition, while socioeconomic status (SES) has been recognized as an important prognostic factor for MM, some of the studies were not able to investigate the effects of SES on MM survival disparities.

Here, we analyze the SEER-Medicare linked database to examine racial disparities in MM survival and associated factors. Instead of the conventional model-based analysis, we used a novel tapered matching approach [13–16] to compare the entire population of NHB patients in the SEER-Medicare database with four matched NHW populations. We sought to study whether NHW patients with MM who present similar to NHB patients with MM receive similar myeloma therapies as NHBs, and if not, to what extent treatment differences explain the disparities in survival.

## Methods

### Study population

**Fig. 1:**
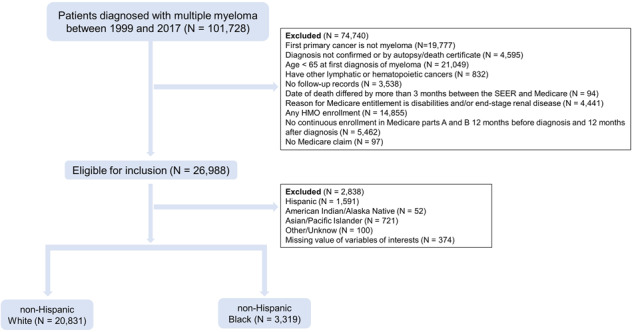
Patient selection. Study cohort selection flow diagram in SEER-Medicare datasets.

This study was approved by the Institutional Review Board at the Medical College of Wisconsin. We investigated patients diagnosed between 1999 and 2017 with MM in the SEER-Medicare database (2020 release), defined by International Classification of Diseases for Oncology, Third Edition using topography codes (C42.1) and histologic codes (M9732/3). Eligible patients must have had continuous enrollment in Medicare parts A and B from 12 months before MM diagnosis to at least 12 months after MM diagnosis or death, whichever occurs first. Patients were excluded if they had duplicate or incomplete records such as death certificate or autopsy cases, and if they enrolled in the health maintenance organization. Patients having other lymphatic or hematopoietic cancers recorded in the database at any time were also excluded. We used the race/ethnicity data in the SEER-Medicare database to define NHWs and NHBs (Fig. 1).

### Variables of Interest

Demographic factors included age at diagnosis, sex, year of diagnosis, SEER site, and marital status. A census tract-level SES score was computed based on neighborhood poverty, income, and education level [14]. Comorbid conditions were combined to generate an individual’s cancer-specific NCI Comorbidity Index [17]. Treatment variables included use of traditional chemotherapy (melphalan, doxorubicin, vincristine, cyclophosphamide, etoposide, bendamustine, and carmustine), PIs (bortezomib, carfilzomib and ixazomib), IMiDs (thalidomide, lenalidomide and pomalidomide) and ASCT.

### Statistical analysis

The outcomes of interest were median OS and the 3-, 5- and 10-year observed survival rates (%) from the diagnosis of MM. Patients were followed from their MM diagnosis date until death from any cause, maximum claim date, or December 31, 2018, to allow for a minimum of 1 year of follow-up evaluation.

A sequential matching process was conducted using propensity score matching (PSM) as described previously [13–16]. Briefly, all NHBs were included in each match and the NHW comparator population changed according to the four matching criteria, based on demographic-, SES-, presentation-, and treatment-related factors, respectively. In the demographics match, we matched NHWs to NHBs by age at diagnosis (matching by minimizing age difference), sex, year of diagnosis (±5 years), SEER site, and marital status. In the SES match, we matched NHWs to NHBs by SES and demographic variables. In the presentation match, we matched NHWs to NHBs by comorbidity score, plus demographic and SES variables. In the treatment match, we matched NHWs to NHBs by treatment variables (traditional chemotherapy, PIs, IMiDs and ASCT), plus demographics, SES, and presentation variables. PSM is a statistical matching technique that attempts to reduce the bias caused by confounders in an estimate of the treatment effect obtained from simply comparing outcomes between patients received and not received the treatment. For example, in the treatment match, the PSM uses logistic regression to regress demographics, SES or comorbidities on race (NHB or NHW) and obtains predicted probabilities for each NHB or NHW patient. Then, we calculate the distances between one NHB patient and all other treatment-matched NHW patients, defined as the absolute value of difference of two predicted probabilities, one from the NHB and the other from an NHW. Finally, we take the minimum distance to find the NHW patient who best matches the NHB patient. This matching process would remove the overlapping controls using the exterior match and allow us to understand the nature of the disparity. The quality of the matching was verified by comparing the similarities of matching variables between NHBs and NHWs using standardized differences in means before and after matching. We considered standardized difference <0.1 standard deviation (SD) as successfully matched. Kaplan-Meier method was used to calculate the median survival and 3-, 5-, and 10-year survival rates, and Log-rank test was used to compare the survival between NHBs and NHWs. We used the bootstrap method to obtain confidence interval for the paired differences in survival and paired Cox proportional hazards models to examine survival over time and hazard ratios (HRs) [18]. All analyses were conducted in SAS 9.4 (SAS Institute, Cary, NC). Statistical significance was determined at *α* = 0.05, and all *P* values for statistical significance were two-sided.

## Results

### Overall matching results

We identified a total of 24,150 patients who were newly diagnosed with MM from 1999 to 2017 and met the inclusion criteria (Fig. 1). Among them, 3319 (13.7%) were NHBs and 20,831 (86.3%) were NHWs. Overall, compared to unmatched NHWs, NHBs were younger on average (76.1 vs.77.1 years; *P* < 0.001), more likely to be female (60.0% vs. 48.0%, *P* < 0.001) and to be unmarried at diagnosis (42.0% vs. 25.2%, *P* < 0.001). NHBs also had lower SES (60.9% vs. 23.1%, *P* < 0.001), but more comorbidities (NCI Comorbidity Index ≥1, 84.9% vs. 78.0%, *P* < 0.001) than NHWs (Table 1).

**Table 1:** Characteristics of non-Hispanic black and non-Hispanic white patients.

Variable	Black patients (*n* = 3319)	Non-Hispanic white patients, n (%)				
		Treatment-matched (*n* = 3319)	Presentation-matched (*n* = 3319)	SES-matched (*n* = 3319)	Demographics-matched (*n* = 3319)	All Whites-unmatched (*n* = 20,831)
*Mean diagnosis year (SD)*	2008.5 (5.09)	**2008.2 (5.14)**	2008.6 (5.15)	**2008.1 (5.08)**	2008.5 (4.95)	2008.4 (5.14)
*Mean age at diagnosis (SD), y*	76.1 (6.88)	76.2 (6.62)	76.2 (6.66)	76.1 (6.59)	76.1 (6.84)	**77.1 (6.93)**
*Female*	1992 (60.02)	1972 (59.42)	1935 (58.30)	1935 (58.30)	1992 (60.02)	**9992 (47.97)**
Marital status						
Married	963 (29.01)	969 (29.20)	959 (28.89)	1025 (30.88)	963 (29.01)	**8376 (40.21)**
Not married	1394 (42.00)	1370 (41.28)	1365 (41.13)	1325 (39.92)	1394 (42.00)	**5255 (25.23)**
Unknown	962 (28.98)	980 (29.53)	995 (29.98)	969 (29.20)	962 (28.98)	**7200 (34.56)**
SES						
Low	2020 (60.86)	2081 (62.70)	2046 (61.65)	2020 (60.86)	**932 (28.08)**	**4809 (23.09)**
Moderate	965 (29.08)	920 (27.72)	949 (28.59)	965 (29.08)	**1605 (48.36)**	**10,062 (48.30)**
High	334 (10.06)	318 (9.58)	324 (9.76)	334 (10.06)	**782 (23.56)**	**5960 (28.61)**
Charlson comorbidity score						
0	502 (15.13)	460 (13.86)	502 (15.13)	**664 (20.01)**	**706 (21.27)**	**4576 (21.97)**
1–2	1053 (31.73)	1121 (33.78)	1053 (31.73)	**1217 (36.67)**	**1243 (37.45)**	**7738 (37.15)**
≥3	1764 (53.15)	1738 (52.37)	1764 (53.15)	**1438 (43.33)**	**1370 (41.28)**	**8517 (40.89)**
Chemotherapy						
No	3002 (90.45)	3002 (90.45)	2954 (89.00)	2985 (89.94)	2970 (89.48)	18,616 (89.37)
Yes	317 (9.55)	317 (9.55)	365 (11.00)	334 (10.06)	349 (10.52)	2215 (10.63)
PIs						
No	2381 (71.74)	2381 (71.74)	**2217 (66.80)**	**2293 (69.09)**	**2235 (67.34)**	**14,142 (67.89)**
Yes	938 (28.26)	938 (28.26)	**1102 (33.20)**	**1026 (30.91)**	**1084 (32.66)**	**6689 (32.11)**
IMiDs						
No	2776 (83.64)	2776 (83.64)	**2636 (79.42)**	**2676 (80.63)**	**2611 (78.67)**	**16,722 (80.27)**
Yes	543 (16.36)	543 (16.36)	**683 (20.58)**	**643 (19.37)**	**708 (21.33)**	**4109 (19.73)**
ASCT						
No	3194 (96.23)	3194 (96.23)	**3124 (94.12)**	**3111 (93.73)**	**3107 (93.61)**	**19,726 (94.70)**
Yes	125 (3.77)	125 (3.77)	**195 (5.88)**	**208 (6.27)**	**212 (6.39)**	**1105 (5.30)**

Note: Variables controlled in some of the four matches but allowed to vary naturally in other matches. The “Black patients” column reports the statistical numbers for all non-Hispanic black patients in the dataset. The “Treatment-matched” column reports the statistical numbers for the closest non-Hispanic white match, namely the treatment match (which also controls for presentation, SES, and demographic variables); the “Presentation-matched” column also controls for SES and demographic variables; the “SES-matched” column also controls for demographic variables. The “All Whites-unmatched” column reports data for all non-Hispanic whites in the dataset without matching. Results for each variable that appear to the left of the bold vertical line are for variables included in the match designated by the column. Results to the right of the bold vertical line are for variables not used in the match designated by the column. Percentages or rates bolded imply statistically significant (*P* < 0.05) differences between non-Hispanic blacks and non-Hispanic whites.

A complete matching table is provided in Supplementary Table 1. In each match, the controlled variables had standardized differences <0.1 SDs, demonstrating successful matches. By design, the four matches sequentially removed some aspects of the racial disparity. The remaining aspects (i.e., unmatched variables) reveal differences that allow us to understand whether these differences contribute to survival disparities.

### Treatment disparity in the overall population

Compared with demographics-matched NHWs, NHBs were less likely to use PIs (28.3% vs. 32.7%, *P* < 0.001), IMiDs (16.4% vs. 21.3%, *P* < 0.001), and ASCT (3.8% vs. 6.4%, *P* < 0.001). The disparities in receipt of effective antimyeloma treatments remained significant even after matching on SES (PIs: 28.3% vs. 30.9%, IMiDs: 16.4% vs. 19.4%, and ASCT: 3.8% vs. 6.3%; all *P* < 0.001) and presentation factors (PIs: 28.3% vs. 33.2%, IMiDs: 16.4% vs. 20.6%, and ASCT: 3.8% vs. 5.9%; all *P* < 0.001). However, there were no differences between NHBs and NHWs in the receipt of traditional chemotherapy (Table 1).

To understand the reason for treatment disparity, we performed regression analysis to identify factors that were associated with the receipt of antimyeloma treatments in the presentation-matched pairs. We found that in addition to race/ethnic, year of diagnosis, age at diagnosis, sex, marital status, SEER site, and SES were also associated with receipt of treatments (Supplementary Table 2).

### Survival disparity in the overall population

**Fig. 2:**
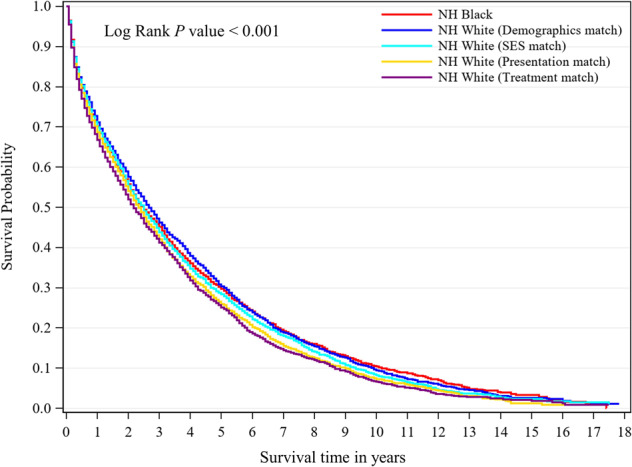
Survival curve. Life-Table plot for multiple myeloma survival for the total non-Hispanic black study population (*n* = 3319) and the three matched non-Hispanic white populations (each *n* = 3319) diagnosed between 1999 and 2017.

During follow-up, 16,479 of 20,831 NHWs (79.1%) and 2595 of 3319 NHBs (78.2%) died. The median survival time was similar in NHBs compared with demographics-matched NHWs (30.0 vs. 32.0 months; *P* = 0.61). The sequential match on SES, presentation and treatment resulted in sequential reductions in the median survival time in NHWs (32 months to 30 months to 28 months to 26 months), with the median survival turning significantly longer for NHBs compared with NHWs in the presentation match (*P* < 0.001) and treatment match (*P* < 0.001) (Table 2, Fig. 2). Likewise, there was no difference in the 5-year survival between NHBs and demographics-matched NHWs (5-year survival difference for NHWs vs. NHBs, 0.6%; *P* = 0.30). The 5-year survival difference flipped to −1.4% but remained non-significant after SES matching (*P* = 0.17) however became statistically significantly longer in NHBs after presentation matching (5-year survival difference, −3.8%; *P* = 0.003). Matching on treatment further increased the 5-year survival difference to −4.6% (*P* < 0.001) (Table 2). Similar patterns of survival disparity changes over sequential matching were also observed in 3- and 10-year survival rates.

**Table 2:** Outcomes of non-Hispanic black and non-Hispanic white patients with multiple myeloma.

Outcome measure	Black patients (*n* = 3319)	Matched non-Hispanic White patients				
		Treatment-matched (*n* = 3319)	Presentation-matched (*n* = 3319)	SES-matched (*n* = 3319)	Demographics-matched (*n* = 3319)	All Whites-unmatched (*n* = 20,831)
Survival, median (95% CI), mo.	30.0 (28.0–32.0)	26.0 (25.0–29.0)	28.0 (26.0–30.0)	30.0 (28.0–31.0)	32.0 (30.0–34.0)	31.0 (30.0–31.0)
* P* value		**<0.001**	**<0.001**	0.139	0.609	0.139
Deaths, n	2595	2731	2659	2690	2614	16479
3-y survival, % (95% CI)	45.1 (43.3–46.8)	41.2 (39.5–43.0)	42.1 (40.3–43.8)	43.8 (42.0–45.5)	46.3 (44.5–48.0)	44.9 (44.2–45.6)
Survival difference, % (95% CI)^a^	NA	−3.8 (−6.5–−1.3)	−3.0 (−5.5–−0.6)	−1.3 (−3.7–1.3)	1.2 (−1.3–3.8)	−0.1 (−2.1–1.7)
* P* value		**<0.001**	**0.009**	0.157	0.321	0.555
Deaths, n	1726	1851	1811	1770	1692	10786
5-y survival, % (95% CI)	29.7 (28.0–31.4)	25.1 (23.5–26.7)	25.9 (24.3–27.6)	28.4 (26.7–30.0)	30.3 (28.6–32.0)	28.2 (27.5–28.9)
Survival difference, % (95% CI)^a^	NA	−4.6 (−7.2–−2.3)	−3.8 (−6.0–−1.4)	−1.4 (−3.8–1.0)	0.6 (−1.9–3.0)	−1.5 (−3.3–0.1)
* P* value		**<0.001**	**0.003**	0.167	0.305	0.329
Deaths, n	2129	2284	2233	2188	2120	13530
10-y survival, % (95% CI)	10.5 (9.1–11.8)	6.6 (5.5–7.7)	7.3 (6.1–8.5)	8.2 (7.0–9.4)	9.5 (8.2–10.8)	8.4 (7.9–8.9)
Survival difference, % (95% CI)^a^	NA	−3.8 (−5.6–−2.0)	−3.2 (−5.1–−1.3)	−2.2 (−4.0–−0.5)	−1.0 (−2.7–0.9)	−2.0 (−3.4–−0.7)
* P* value		**<0.001**	**0.001**	0.126	0.555	0.144
Deaths, n	2515	2672	2600	2613	2543	16044
Paired Cox model, HR (95% CI)	Ref.	1.17 (1.09–1.26)	1.09 (1.01–1.17)	1.03 (0.96–1.11)	0.95 (0.88–1.02)	1.03 (0.99–1.07)
* P* value		**<0.001**	**0.025**	0.359	0.144	0.146

Demographics indicates matching of non-Hispanic black and non-Hispanic white patients on age at diagnosis, sex, year of diagnosis, SEER site and marital status.

SES indicates matching on demographic variables plus SES.

Presentation indicates matching on demographic variables, SES, plus comorbid conditions.

Treatment indicates matching on demographic, SES and presentation plus chemotherapy, PIs, IMiDs, and ASCT.

^a^Survival differences between non-Hispanic blacks and matched non-Hispanic white patients. Confidence Intervals (CIs) were calculated by 1000 bootstrap resampling.

*P* values bolded imply statistically significant (*P* < 0.05).

**Fig. 3:**
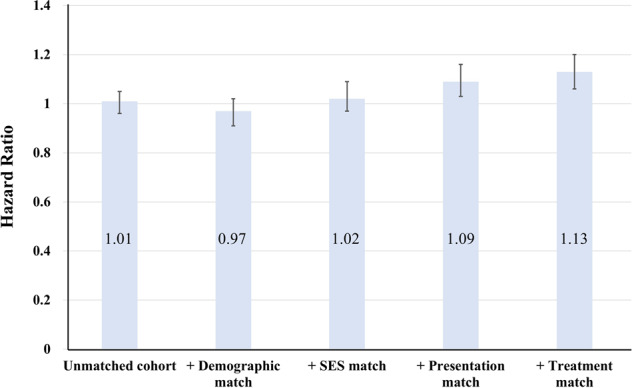
HRs in four matches. HR of all-cause 5-year mortality risk for sequentially matched non-Hispanic whites vs. non-Hispanic blacks.

The results from the Cox regression analysis mirrored those of the matching approach (Fig. 3). Compared with NHBs, NHWs had a 2% (HR, 1.02; 95% CI, 0.97–1.09, *P* = 0.36) excess risk of all-cause 5-year mortality in the SES match. The excess risk was increased to 9% (HR, 1.09; 95% CI, 1.03–1.16, *P* = 0.03) after presentation matching, and to 13% (HR, 1.13; 95% CI, 1.06–1.20, *P* < 0.001) after treatment matching (Fig. 3).

### Survival disparity by SES

We investigated the effects of presentation and treatment-related factors on survival disparity stratified by SES. Because 60% of NHBs had low SES, we merged moderate and high SES into a single group to improve power. Overall, patients with higher SES had better survival than patients with lower SES. For patients with low SES, the differences in survival were not statistically significant in the demographics and presentation match. Further matching on treatment substantially increased the survival disparity, with NHBs having a significantly longer 5-year survival rate than treatment-matched NHWs (absolute difference, 2.6%; *P* = 0.03) (Supplementary Table 3). Similar patterns were also observed for 3-, and 10-year survival rates. Among patients with moderate/high SES, the results were similar as the main results (Supplementary Table 3).

### Change in survival disparity over time

We then investigated the change in survival disparity over time. Because MM survival was greatly improved after introduction of two novel class of agents, the PIs and IMiDs, we used the year of diagnosis in 2003 and 2007 as cut off to separate patients to three groups: diagnosis before 2003 (2000–2002), diagnosis before 2008 (2003–2007, introduction of thalidomide in induction), and diagnosis in 2008 and later (2008–2017, introduction of bortezomib and lenalidomide induction), similar to the approach used in the study by Costa et al. [6]. The change in survival disparity over time is shown in Supplementary Table 4. As expected, survival rates for NHB and NHW patients with MM improved over time, suggesting the effectiveness of the use of novel agents in antimyeloma treatment. The racial survival disparity was not significant across all analysis in patients diagnosed before 2003, when chemotherapy remained the main treatment for MM, which likely reflected the factor that no difference in chemotherapy was observed in our study and small sample size in this stratum. For patients diagnosed between 2003 and 2007, when novel agents became clinically available, the difference in survival rates between NHB and NHW patients was significant only in the treatment match, with the 3-, 5- and 10-year survival disparity of 4.8%, 4.6% and 5.2%, respectively (all *P* < 0.01). For patients diagnosed in 2008 and later, by when 75% of newly diagnosed patients in the US received one of the new agents as part of their initial therapy, the racial survival difference was marginally significant in the treatment match, indicating that the widespread use of novel agents helped to reduce the racial survival disparity (Supplementary Table 4).

## Discussion

In a matched cohort study using the most recent data from SEER-Medicare (2020 release), we found that NHBs were less likely to receive novel therapies and ASCT than NHWs even among patients matched for demographics, SES, and comorbidities. Despite the survival time was comparable between NHB and unmatched NHW patients with MM, NHBs exhibited significantly longer OS than NHWs when they were treated similar, reinforcing the importance of equitable access to effective treatment modalities to improve the survival outcomes of NHB patients with MM and highlight the need for further studies to elucidate reasons for racial differences in comorbidities. This also suggests that NHBs are likely enriched with a higher proportion of good biologic risk patients.

Evolution of treatment to include PIs, IMiDs, and ASCT has led to a doubling of OS in MM over the past decade [19–21]. However, this survival benefit has been experienced primarily by NHW patients, which was believed to be due to the differences in utilization of MM treatment [4, 6–8, 10, 22–26]. A retrospective analysis using SEER-Medicare data between 2000 and 2011 showed NHBs were 37% less likely to undergo ASCT and 21% less likely to use bortezomib than NHWs [7]. Using data from the Center for International Blood and Marrow Transplant Research (CIBMTR), our group also observed a significantly lower utilization rate of ASCT in NHB patients with MM (12.2% in 2008–20.5% in 2013) compared with NHW patients (22.6% in 2008–37.8% in 2013) [22]. Similar results were reported by others using trial data and registry dataset [8, 10, 26]. Consistent with previous studies, our analysis showed that NHBs were less likely to receive PIs, IMiDs and ASCT than NHWs. In addition, we showed this treatment disparity persisted even when NHWs presented at diagnosis similar to NHBs patients (i.e., presentation match), indicating SES and comorbidities could not account for treatment disparities between NHB and NHW patients with MM. Other barriers, such as social and cultural beliefs, underrepresentation of minorities in clinical trials, referral bias and social support, may contribute to the racial disparity in MM treatment. Because treatment is an important modifiable factor, addressing these treatment barriers in NHBs is critical to improve patient survival in underserved populations. In our study, about 4% of NHBs and 6% of NHWs used ASCT, which are similar as previous reports from SEER-Medicare [7, 8]. However, due to variations in methodology, such as the selection of agents included in the treatment variables, direct comparisons for the utilization rates of IMiDs, PIs and chemotherapy with other studies are difficult. In addition, the major goal of the current study is to assess racial differences in MM survival, and the sequential effects of patient characteristics, and diagnosis and treatment-related factors on the survival disparity, we therefore did not evaluate the survival differences among treatment options. It is possible that some of the agents are more effective in certain racial groups than others, which requires further investigations.

Using tapered matching approach, studies in breast, colon and esophageal cancers found that NHBs had significantly inferior survival than demographics-matched NHWs and the racial survival disparity was reduced but remained significant after further matching on presentation [14, 15, 27–29]. Unlike these studies, the racial survival differences in MM were not statistically significant when matching on demographic variables. In sharp contrast, after adequately matching on presentation, NHBs had significantly longer survival than NHWs, suggesting the associations between increased number of comorbidities in NHBs and decreased survival [30]. The racial survival disparities were further enlarged after sequential matching on treatment which confirmed the effects of the observed treatment disparity on the survival disparity even among patients matched for presentation. Although the superior survival observed among NHB patients compared with presentation- and treatment-matched NHWs is rarely seen in other malignancies, our findings are in line with previous reports. For example, initial SEER-based studies showed that NHBs had significantly better OS and/or myeloma-specific survival than NHWs [4, 9]. An analysis of SEER-Medicare 2000–2011 database with 20,916 MM patients found NHBs had 9% increased survival than that of NHWs when controlling for demographics, income, comorbidities, and treatment use [7]. A longer OS for NHBs was also reported among patients undergoing ASCT in the Connect MM registry [31]. The superior survival observed in NHBs with MM may reflect biological heterogeneity of the disease among racial groups. An investigation of racial differences in cytogenetic abnormalities found NHBs had significantly lower frequency of “high-risk” MM cytogenetic abnormalities *t*(4;14) and del(17/17p) than NHWs [32]. Sequencing based analysis reported NHBs had a lower prevalence of *TP53* mutations compared with NHWs [33, 34]. The presence of *TP53* mutations typically confers significantly worse OS of MM [35, 36]. This evidence suggests NHBs may harbor a more indolent disease subtype than NHWs. We also noted reports that were inconsistent with current analysis. For instance, our previous study in the CIBMTR focusing on recipients of ASCT for MM found NHBs had similar outcomes compared to NHWs [22, 23] though recent studies have shown superior post-transplant survival in NHBs with *t*(11;14) compared to NHWs [37]. In a retrospective study of 15,717 MM patients from the Veterans Affairs (VA) system, where patients had equal access to treatment, NHBs and NHWs had similar OS among patients aged 65 years or older—the same age group of patients as our current study, despite significantly superior OS was observed for patients <65 years old [11]. A similar OS was also found in studies using SEER-Medicare 2007–2013 database, the Cooperative Group clinical trial data and the National Cancer Database [8, 38, 39]. In contrast, in the CoMMpass study, where the utilization rate of novel agents and ASCT are both higher than other studies, NHBs had significantly inferior OS than NHWs and that this risk was only partly abrogated by the receipt of treatment [10]. However, direct comparison across studies is challenging due to the differences in study populations, treatment use, and covariate adjustments.

Our matching strategy to include SES, a well-recognized prognostic factor of MM, provided us with important clues as to the effects of SES on racial survival disparities. We confirmed that lower SES was associated with shorter OS in both racial groups. When NHWs and NHBs were matched for SES variables (i.e., SES match), their OS were comparable. Further stratified analysis by SES showed that among patients with low SES, the significantly longer OS for NHBs was only observed in the treatment-matched cohorts, but not in the presentation match. However, among patients with moderate/high SES, presentation and treatment still had significant effects on racial survival disparity, which was highly consistent with the results derived from the complete cohort. Together, these findings indicate that the effects of treatment difference in survival disparity by race may be independent of SES.

A strength of our study is the use of novel minimum distance matching strategy. Most studies used model-based methods to explain racial disparities which, when NHWs make up the majority of population, the model coefficients may reflect the NHW population. Compared with model-based analyses, tapered matching allowed us to investigate the influence of patients-, disease- and treatment-related factors each on the racial disparities in MM survival. Given the large number of NHWs available in the SEER-Medicare database (*n* = 20,831), we were able to achieve very close matches with 3319 NHB patients. We found treatment utilization as the most important contributor to superior survival among NHBs. More importantly, we found that even when NHWs present similar to NHBs with MM, NHB patients still had lower utilization of novel agents and ASCT than NHWs. The reasons for differences in receipt of treatment are multifactorial, likely involving social, clinical, and host factors that need further investigation.

There are several limitations in our study. First, treatment definitions were based on claims, misclassification is possible without verification from chart review. Second, the data do not capture all relevant factors for MM survival, for example, performance status, cytogenetic abnormalities, and person’s willingness to receive treatment, such as cultural beliefs and values. By design, our study population was restricted to patients covered by Medicare, thus the impact of access to care could not be addressed. Third, NHB patients have an average earlier age of onset of MM than NHWs (65 vs. 70 years old) [4], while the SEER-Medicare database was limited to patients ≥ 65 years old at diagnosis and only represents about 48% of the U.S. population with some of the states fully covered (e.g., HI, CA, UT, NM, IA, KY, GA, CT, NJ, and LA), making it problematic to generalize our findings in younger patents that have greater representation in the NHB group. In addition, SES was measured at the census tract level instead of at the individual level. It is possible that SES may contribute more to the racial disparity in MM survival and receipt of treatment among younger populations. Furthermore, because of the nature of the matching process and the circumstance that over a quarter of patients in our dataset lack information on disease-specific mortality, we were not able to assess myeloma-specific mortality in this study.

In summary, in the SEER-Medicare population, NHBs with MM were less likely to receive novel antimyeloma treatment and ASCT compared with NHWs, and these disparities in treatment could not be explained by sociodemographic factors. Although the OS was comparable between NHBs and NHWs across the entire population, NHBs had a superior survival when they were treated similar as NHWs. Future research should explore the biological mechanisms for the differences in comorbidities and implications for treatment.

## Supplementary information

Supplementary Tables
