# Supplementary material for: Black patients with multiple myeloma have better survival than white patients when treated equally: a matched cohort study
Source: Blood Cancer J. 2022 Feb 24;12(2):34. doi: 10.1038/s41408-022-00633-5 (PMC8873507; doi:10.1038/s41408-022-00633-5)
Supplement: Supplementary file 1 — Supplementary Tables [file 41408_2022_633_MOESM1_ESM.docx]

**Supplementary Table 1. Detailed Overall Patient Characteristics and Matching Quality**

| **Variable** | | **Black patients**  **(n=3,319)** | **Non-Hispanic White Patients, n (%)** | | | | | | | |
| --- | --- | --- | --- | --- | --- | --- | --- | --- | --- | --- |
|  |  |  | **Treatment- matched** | | **Presentation-matched** | | **SES-matched** | | **Demographics- matched** | |
|  |  |  | **(n=3,319)** | **SDD** | **(n=3,319)** | **SDD** | **(n=3,319)** | **SDD** | **(n=3,319)** | **SDD** |
| **Mean age at diagnosis (SD), y** | | 76.1 (6.88) | 76.2 (6.62) | -0.02 | 76.2 (6.66) | -0.03 | 76.1 (6.59) | -0.01 | 76.1 (6.84) | 0.00 |
| **Mean diagnosis year (SD)** | | 2008.5 (5.09) | 2008.2 (5.14) | 0.06 | 2008.6 (5.15) | -0.01 | 2008.1 (5.08) | 0.07 | 2008.5 (4.95) | 0.00 |
| **Female** | | 1,992 (60.02) | 1,972 (59.42) | 0.01 | 1,935 (58.30) | 0.03 | 1,935 (58.30) | 0.03 | 1,992 (60.02) | 0.00 |
| **SEER registry site region** | | |  |  |  |  |  |  |  |  |
|  | New England | 194 (5.85) | 188 (5.66) | 0.01 | 176 (5.30) | 0.02 | 169 (5.09) | 0.03 | 194 (5.85) | 0.00 |
|  | Middle Atlantic | 1,107 (33.35) | 1,088 (32.78) | 0.01 | 1,113 (33.53) | 0.00 | 1,005 (30.28) | 0.07 | 1,107 (33.35) | 0.00 |
|  | North Central | 422 (12.71) | 369 (11.12) | 0.05 | 373 (11.24) | 0.05 | 327 (9.85) | 0.09 | 422 (12.71) | 0.00 |
|  | South Atlantic | 606 (18.26) | 600 (18.08) | 0.00 | 616 (18.56) | -0.01 | 612 (18.44) | 0.00 | 606 (18.26) | 0.00 |
|  | South Central | 571 (17.20) | 623 (18.77) | -0.04 | 607 (18.29) | -0.03 | 771 (23.23) | -0.15 | 571 (17.20) | 0.00 |
|  | Mountain/Pacific | 419 (12.62) | 451 (13.59) | -0.03 | 434 (13.07) | -0.01 | 435 (13.11) | 0.00 | 419 (12.62) | 0.00 |
| **Marital status** | |  |  |  |  |  |  |  |  |  |
|  | Married | 963 (29.01) | 969 (29.20) | 0.00 | 959 (28.89) | 0.00 | 1,025 (30.88) | -0.04 | 963 (29.01) | 0.00 |
|  | Not married | 1,394 (42.00) | 1,370 (41.28) | 0.01 | 1,365 (41.13) | 0.02 | 1,325 (39.92) | 0.04 | 1,394 (42.00) | 0.00 |
|  | Unknown | 962 (28.98) | 980 (29.53) | -0.01 | 995 (29.98) | -0.02 | 969 (29.20) | 0.00 | 962 (28.98) | 0.00 |
| **SES** | |  |  |  |  |  |  |  |  |  |
|  | Low | 2,020 (60.86) | 2,081 (62.70) | -0.04 | 2,046 (61.65) | -0.02 | 2,020 (60.86) | 0.00 | 932 (28.08) | 0.70 |
|  | Moderate | 965 (29.08) | 920 (27.72) | 0.03 | 949 (28.59) | 0.01 | 965 (29.08) | 0.00 | 1,605 (48.36) | -0.40 |
|  | High | 334 (10.06) | 318 (9.58) | 0.02 | 324 (9.76) | 0.01 | 334 (10.06) | 0.00 | 782 (23.56) | -0.37 |
| **Charlson comorbidity score** | | |  |  |  |  |  |  |  |  |
|  | 0 | 502 (15.13) | 460 (13.86) | 0.04 | 502 (15.13) | 0.00 | 664 (20.01) | -0.13 | 706 (21.27) | -0.16 |
|  | 1-2 | 1,053 (31.73) | 1,121 (33.78) | -0.04 | 1,053 (31.73) | 0.00 | 1,217 (36.67) | -0.10 | 1,243 (37.45) | -0.12 |
|  | >=3 | 1,764 (53.15) | 1,738 (52.37) | 0.02 | 1,764 (53.15) | 0.00 | 1,438 (43.33) | 0.20 | 1,370 (41.28) | 0.24 |
| **Chemotherapy** | |  |  |  |  |  |  |  |  |  |
|  | No | 3,002 (90.45) | 3,002 (90.45) | 0.00 | 2,954 (89.00) | 0.05 | 2,985 (89.94) | 0.02 | 2,970 (89.48) | 0.03 |
|  | Yes | 317 (9.55) | 317 (9.55) | 0.00 | 365 (11.00) | -0.05 | 334 (10.06) | -0.02 | 349 (10.52) | -0.03 |
| **PIs** | |  |  |  |  |  |  |  |  |  |
|  | No | 2,381 (71.74) | 2,381 (71.74) | 0.00 | 2,217 (66.80) | 0.11 | 2,293 (69.09) | 0.06 | 2,235 (67.34) | 0.10 |
|  | Yes | 938 (28.26) | 938 (28.26) | 0.00 | 1,102 (33.20) | -0.11 | 1,026 (30.91) | -0.06 | 1,084 (32.66) | -0.10 |
| **IMiDs** | |  |  |  |  |  |  |  |  |  |
|  | No | 2,776 (83.64) | 2,776 (83.64) | 0.00 | 2,636 (79.42) | 0.11 | 2,676 (80.63) | 0.08 | 2,611 (78.67) | 0.13 |
|  | Yes | 543 (16.36) | 543 (16.36) | 0.00 | 683 (20.58) | -0.11 | 643 (19.37) | -0.08 | 708 (21.33) | -0.13 |
| **ASCT** | |  |  |  |  |  |  |  |  |  |
|  | No | 3,194 (96.23) | 3,194 (96.23) | 0.00 | 3,124 (94.12) | 0.10 | 3,111 (93.73) | 0.11 | 3,107 (93.61) | 0.12 |
|  | Yes | 125 (3.77) | 125 (3.77) | 0.00 | 195 (5.88) | -0.10 | 208 (6.27) | -0.11 | 212 (6.39) | -0.12 |

Note: Variables controlled in some of the 4 matches but allowed to vary naturally in other matches. The “Black patients” column reports the statistical numbers for all non-Hispanic black patients in the data set. The “Treatment-matched” column reports the statistical numbers for the closest non-Hispanic white match, namely the treatment match (which also controls for presentation, SES, and demographic variables); the “Presentation-matched” column also controls for SES and demographic variables; the “SES-matched” column also controls for demographic variables. The “All Whites-unmatched” column reports data for all non-Hispanic whites in the data set without matching. Results for each variable that appear to the left of the bold vertical line are for variables included in the match designated by the column. Results to the right of the bold vertical line are for variables not used in the match designated by the column. Percentages or rates bolded imply statistically significant (*P* < 0.05) differences between non-Hispanic blacks and non-Hispanic whites.

**Supplementary Table 2. Regression analysis for not receiving treatments in pairs matched on presentatio****n.**

| **Predictor** | **Univariate model** | | **Multivariable model** | |
| --- | --- | --- | --- | --- |
|  | **OR (95% CI)** | ***P*** | **OR (95% CI)** | ***P*** |
|  | | | | |
| **Year of diagnosis** | 0.82 (0.81 - 0.83) | <.001 | 0.81 (0.80 - 0.82) | <.001 |
| **SEER site** |  | <.001 |  | 0.001 |
| New England | Ref |  | Ref |  |
| Middle Atlantic | 1.41 (1.13 - 1.76) |  | 1.61 (1.25 - 2.07) |  |
| North Central | 1.12 (0.87 - 1.43) |  | 1.18 (0.88 - 1.58) |  |
| South Atlantic | 1.04 (0.83 - 1.32) |  | 1.49 (1.13 - 1.97) |  |
| South Central | 1.14 (0.90 - 1.44) |  | 1.32 (0.99 - 1.75) |  |
| Mountain | 1.32 (0.50 - 3.48) |  | 1.18 (0.38 - 3.60) |  |
| Pacific | 1.00 (0.78 - 1.27) |  | 1.23 (0.92 - 1.65) |  |
| **Age at diagnosis** | 1.06 (1.05 - 1.07) | <.001 | 1.07 (1.06 - 1.08) | <.001 |
| **Sex** |  | 0.06 |  | 0.02 |
| Male | Ref |  | Ref |  |
| Female | 1.10 (1.00 - 1.22) |  | 0.86 (0.77 - 0.97) |  |
| **Marital status** |  | <.001 |  | 0.001 |
| Married | Ref |  | Ref |  |
| Not married | 1.40 (1.25 - 1.58) |  | 1.32 (1.15 - 1.52) |  |
| **SES** |  | <.001 |  | 0.10 |
| Low | Ref |  | Ref |  |
| Moderate | 0.77 (0.69 - 0.86) |  | 0.96 (0.84 - 1.09) |  |
| High | 0.50(0.43 - 0.59) |  | 0.80 (0.66 - 0.98) |  |
| **Charlson comorbidity** | | 0.67 |  | 0.53 |
| 0 | Ref |  | Ref |  |
| 1-2 | 1.06 (0.91 - 1.23) |  | 1.02 (0.86 - 1.22) |  |
| >=3 | 1.07 (0.93 - 1.23) |  | 1.08 (0.92 - 1.28) |  |

**Supplementary Table 3. Outcomes of non-Hispanic black and non-Hispanic white patients by SES**

| **Outcome measure** | | | **Black patients** | **Matched non-Hispanic White patients** | | | |
| --- | --- | --- | --- | --- | --- | --- | --- |
|  |  |  |  | **Treatment-matched** | **Presentation- matched** | | **Demographics- matched** |
| **SES-Low (n = 1954, each)** | | |  |  |  | |  |
|  | **Survival, median (95% CI), mo.** | | 26.0 (25.0 - 28.0) | 25.0 (23.0 - 27.0) | 26.0 (25.0 - 29.0) | | 27.0 (25.0 - 30.0) |
|  |  | *P* value |  | **0.003** | 0.093 | | 0.324 |
|  | **3-y survival, % (95% CI)** | | 41.7 (39.4 - 44.0) | 39.2 (37.0 - 41.5) | 40.4 (38.2 - 42.7) | | 41.8 (39.5 - 44.0) |
|  |  | Survival difference, % (95% CI) ^a^ |  | -2.5 (-5.7 - 0.4) | -1.3 (-4.6 - 1.7) | | 0.0 (-3.0 - 3.4) |
|  |  | *P* value |  | **0.025** | 0.481 | | 0.844 |
|  | **5-y survival, % (95% CI)** | | 26.4 (24.3 - 28.5) | 23.8 (21.8 - 25.8) | 24.1 (22.1 - 26.1) | | 25.3 (23.3 - 27.3) |
|  |  | Survival difference, % (95% CI) ^a^ |  | -2.6 (-5.5 - 0.4) | -2.3 (-5.5 - 0.6) | | -1.1 (-4.1 - 1.8) |
|  |  | *P* value |  | **0.025** | 0.224 | | 0.623 |
|  | **10-y survival, % (95% CI)** | | 8.5 (7.0 - 10.0) | 5.2 (3.9 - 6.4) | 6.6 (5.2 - 8.0) | | 6.9 (5.5 - 8.3) |
|  |  | Survival difference, % (95% CI) ^a^ |  | -3.3 (-5.3 - -1.5) | -1.9 (-3.8 - 0.2) | | -1.6 (-3.6 - 0.5) |
|  |  | *P* value |  | **0.003** | 0.100 | | 0.424 |
|  | **Paired Cox model, HR (95% CI)** | | Ref | 1.13 (1.03 - 1.24) | 1.03 (0.93 - 1.13) | | 0.98 (0.89 - 1.07) |
|  |  | *P* value |  | **0.012** | 0.599 | | 0.652 |
| **SES-Moderate/High (n = 1294, each)** | | |  |  |  |  | |
|  | **Survival, median (95% CI), mo.** | | 37.0 (34.0 - 41.0) | 33.0 (29.0 - 36.0) | 31.0 (29.0 - 35.0) | 35.0 (32.0 - 38.0) | |
|  |  | *P* value |  | **0.018** | **0.015** | 0.089 | |
|  | **3-y survival, % (95% CI)** | | 50.6 (47.7 – 53.5) | 47.1 (44.3 - 50.0) | 45.4 (42.5 - 48.3) | 48.7 (45.8 - 51.6) | |
|  |  | Survival difference, % (95% CI) ^a^ |  | -3.5 (-7.7 - 0.4) | -5.2 (-9.4 - -1.3) | -1.9 (-5.9 - 2.0) | |
|  |  | *P* value |  | **0.017** | **0.012** | 0.160 | |
|  | **5-y survival, % (95% CI)** | | 35.1 (32.2 – 38.0) | 30.0 (27.2 - 32.8) | 30.7 (27.9 - 33.6) | 33.3 (30.5 - 36.1) | |
|  |  | Survival difference, % (95% CI) ^a^ |  | -5.1 (-9.0 - -1.2) | -4.3 (-8.3 - -0.4) | -1.8 (-5.8 - 2.5) | |
|  |  | *P* value |  | **0.010** | **0.013** | 0.282 | |
|  | **10-y survival, % (95% CI)** | | 14.0 (11.3 – 16.7) | 10.3 (8.0 - 12.5) | 10.5 (8.1 - 13.0) | 10.3 (7.9 - 12.6) | |
|  |  | Survival difference, % (95% CI) ^a^ |  | -3.7 (-7.5 - -0.2) | -3.5 (-7.0 - 0.3) | -3.7 (-7.6 - -0.0) | |
|  |  | *P* value |  | **0.018** | **0.009** | 0.118 | |
|  | **Paired Cox model, HR (95% CI)** | | Ref. | 1.10 (0.97 - 1.24) | 1.06 (0.94 - 1.20) | 1.09 (0.97 - 1.23) | |
|  |  | *P* value |  | 0.128 | 0.332 | 0.160 | |

^a^ Survival differences between non-Hispanic blacks and matched non-Hispanic white patients. Confidence Intervals (CIs) were calculated by 1000 bootstrap resampling.

Demographics indicates matching of non-Hispanic black and non-Hispanic white patients on age at diagnosis, sex, year of diagnosis, SEER site and marital status; Presentation indicates matching on demographic variables plus comorbid conditions; Treatment indicates matching on demographics and presentation plus chemotherapy, PIs, IMiDs, and ASCT

**Supplementary Table 4. Outcomes of non-Hispanic black and non-Hispanic white patients by time periods**

| **Outcome measure** | | | **Black patients** |  | **Matched non-Hispanic White patients** | | |
| --- | --- | --- | --- | --- | --- | --- | --- |
|  |  |  |  | **Treatment- matched** | **Presentation-matched** | **SES-matched** | **Demographics- matched** |
| **Diagnosis year 2000-2002 (n = 480, each)** | | | |  |  |  |  |
|  | **Survival, median (95% CI), mo.** | | 25.5 (22.0 - 29.0) | 24.0 (20.0 - 28.0) | 24.0 (20.0-28.0) | 26.0 (22.0 - 29.0) | 24.0 (19.0 - 28.0) |
|  |  | *P* value |  | 0.416 | 0.341 | 0.297 | 0.827 |
|  | **3-y survival, % (95% CI)** | | 38.8 (34.4 - 43.1) | 36.0 (31.7 - 40.3) | 36.7 (32.4 - 41.0) | 36.4 (32.1 - 40.7) | 36.0 (31.7 - 40.3) |
|  |  | Survival difference, % (95% CI) ^a^ |  | -2.7 (-8.6 - 3.3) | -2.1 (-8.1 - 4.3) | -2.4 (-8.5 - 3.9) | -2.7 (-8.6 - 3.2) |
|  |  | *P* value |  | 0.352 | 0.277 | 0.386 | 0.446 |
|  | **5-y survival, % (95% CI)** | | 24.0 (20.1 - 27.8) | 19.0 (15.5 - 22.5) | 19.8 (16.2 - 23.4) | 20.3 (16.7 - 23.9) | 21.0 (17.4 - 24.7) |
|  |  | Survival difference, % (95% CI) ^a^ |  | -5.0 (-10.0 - 0.3) | -4.2 (-9.4 - 1.3) | -3.7 (-8.6 - 1.5) | -2.9 (-8.0 - 2.7) |
|  |  | *P* value |  | 0.207 | 0.231 | 0.302 | 0.433 |
|  | **10-y survival, % (95% CI)** | | 6.3 (4.1- 8.4) | 5.6 (3.6 - 7.7) | 6.0 (3.9 - 8.2) | 5.4 (3.4 - 7.5) | 7.3 (5.0 - 9.6) |
|  |  | Survival difference, % (95% CI) ^a^ |  | -0.6 (-3.6 - 2.5) | -0.2 (-3.3 - 2.9) | -0.8 (-3.8 - 2.0) | 1.0 (-1.9 - 4.5) |
|  |  | *P* value |  | 0.340 | 0.407 | 0.358 | 0.675 |
|  |  |  |  |  |  |  |  |
| **Diagnosis year 2003-2007 (n = 998, each)** | | | |  |  |  |  |
|  | **Survival, median (95% CI), mo.** | | 27.0 (24.0 - 30.0) | 23.0 (20.0 - 25.0) | 25.0 (23.0 - 29.0) | 27.0 (24.0 - 31.0) | 31.0 (26.0 - 34.0) |
|  |  | *P* value |  | **< 0.001** | **0.023** | 0.456 | 0.726 |
|  | **3-y survival, % (95% CI)** | | 42.5 (39.4 - 45.6) | 37.7 (34.7 - 40.7) | 40.8 (37.7 - 43.8) | 43.2 (40.2 - 46.3) | 45.2 (42.1 - 48.3) |
|  |  | Survival difference, % (95% CI) ^a^ |  | -4.8 (-9.0 - -0.2) | -1.7 (-5.8 - 2.8) | 0.8 (-3.7 - 5.3) | 2.7 (-1.7 - 7.3) |
|  |  | *P* value |  | **0.006** | 0.238 | 0.882 | 0.482 |
|  | **5-y survival, % (95% CI)** | | 27.5 (24.7 - 30.3) | 22.9 (20.3 - 25.6) | 24.5 (21.9 - 27.2) | 28.3 (25.5 - 31.1) | 29.8 (26.9 - 32.6) |
|  |  | Survival difference, % (95% CI) ^a^ |  | -4.6 (-8.0 - -0.6) | -3.0 (-6.9 - 0.8) | 0.8 (-3.3 - 4.7) | 2.2 (-1.6 - 6.1) |
|  |  | *P* value |  | **0.004** | 0.142 | 0.975 | 0.494 |
|  | **10-y survival, % (95% CI)** | | 11.8 (9.8-13.8) | 6.6 (5.1 - 8.2) | 7.6 (5.9 - 9.2) | 9.3 (7.5 - 11.1) | 9.3 (7.5 - 11.1) |
|  |  | Survival difference, % (95% CI) ^a^ |  | -5.2 (-7.7 - -2.7) | -4.3 (-6.7 - -1.7) | -2.5 (-5.2 - 0.2) | -2.6 (-5.1 - 0.1) |
|  |  | *P* value |  | **< 0.001** | **0.026** | 0.501 | 0.942 |
|  |  |  |  |  |  |  |  |
| **Diagnosis year 2008-2017 (n =1825, each)** | | | |  |  |  |  |
|  | **Survival, median (95% CI), mo.** | | 34.0 (31.0 - 37.0) | 31.0 (29.0 - 34.0) | 32.0 (30.0 - 35.0) | 34.0 (31.0 - 37.0) | 35.0 (32.0 - 37.0) |
|  |  | *P* value |  | 0.086 | 0.179 | 0.944 | 0.979 |
|  | **3-y survival, % (95% CI)** | | 48.5 (46.0 - 51.0) | 45.5 (43.0 - 48.0) | 46.8 (44.3 - 49.3) | 48.2 (45.7 - 50.6) | 48.2 (45.8 - 50.7) |
|  |  | Survival difference, % (95% CI) ^a^ |  | -3.0 (-6.3 - 0.4) | -1.7 (-5.2 - 1.8) | -0.3 (-3.8 - 3.3) | -0.3 (-4.0 - 3.2) |
|  |  | *P* value |  | 0.064 | 0.197 | 0.892 | 0.769 |
|  | **5-y survival, % (95% CI)** | | 33.0 (30.5 - 35.6) | 30.4 (28.0 - 32.9) | 30.2 (27.7 - 32.7) | 31.9 (29.4 - 34.3) | 30.7 (28.2 - 33.2) |
|  |  | Survival difference, % (95% CI) ^a^ |  | -2.6 (-6.1 - 1.0) | -2.8 (-6.2 - 0.9) | -1.2 (-4.9 - 2.3) | -2.4 (-5.8 - 1.4) |
|  |  | *P* value |  | 0.067 | 0.181 | 0.900 | 0.959 |
|  | **10-y survival, % (95% CI)** | | 6.7 (2.2 - 11.1) | 6.6 (3.8 - 9.4) | 5.4 (2.7 - 8.1) | 5.8 (1.7 - 9.9) | 5.6 (1.7 - 9.4) |
|  |  | Survival difference, % (95% CI) ^a^ |  | -0.0 (-5.4 - 6.1) | -1.2 (-6.7 - 5.0) | -0.8 (-7.5 - 6.0) | -1.1 (-7.4 - 5.4) |
|  |  | *P* value |  | 0.083 | 0.175 | 0.962 | 0.978 |

^a^ Survival differences between non-Hispanic blacks and matched non-Hispanic white patients. Confidence Intervals (CIs) were calculated by 1000 bootstrap resampling.

Demographics indicates matching of non-Hispanic black and non-Hispanic white patients on age at diagnosis, sex, year of diagnosis, SEER site and marital status.

SES indicates matching on demographic variables plus SES.

Presentation indicates matching on demographic variables, SES, plus comorbid conditions.

Treatment indicates matching on demographic, SES and presentation plus chemotherapy, PIs, IMiDs, and ASCT.
